# The role of glutamate transporters in the pathophysiology of neuropsychiatric disorders

**DOI:** 10.1038/s41537-017-0037-1

**Published:** 2017-09-21

**Authors:** Sinead M. O’Donovan, Courtney R. Sullivan, Robert E. McCullumsmith

**Affiliations:** Department of Psychiatry, University of Cincinnati, Cincinnati, OH 45221 USA

## Abstract

Altered glutamate transporter expression is a common feature of many neuropsychiatric conditions, including schizophrenia. Excitatory amino acid transporters (EAATs) are responsible for the reuptake of glutamate, preventing non-physiological spillover from the synapse. Postmortem studies have revealed significant dysregulation of EAAT expression in various brain regions at the cellular and subcellular level. Recent animal studies have also demonstrated a role for glutamate spillover as a mechanism of disease. In this review, we describe current evidence for the role of glutamate transporters in regulating synaptic plasticity and transmission. In neuropsychiatric conditions, EAAT splice variant expression is altered. There are changes in the localization of the transporters and disruption of the metabolic and structural protein network that supports EAAT activity. This results in aberrant neuroplasticity and excitatory signaling, contributing to the symptoms associated with neuropsychiatric disease. Understanding the complex functions of glutamate transporters will clarify the relevance of their role in the pathophysiology of neuropsychiatric disorders.

## Introduction

This review summarizes the essential role of glutamate transporters in preventing spillover of glutamate and the resulting aberrant synaptic plasticity. It provides a synthesis of recent findings implicating glutamate transporters and their splice variants in the pathophysiology of schizophrenia and other neuropsychiatric disorders.

A common characteristic of schizophrenia is a progressive change in brain volume, especially in frontal cortex and thalamic regions.^1,2^ Pathological gray matter loss and the associated circuitry deficits in schizophrenia are driven by impaired neuroplasticity and manifest as a reduction in spine density and dendritic arbors,^3^ rather than a loss of neurons or the gliosis that is associated with neurodegenerative disease.^4^ Interestingly, neurocognitive decline in schizophrenia is seen before other symptoms arise in neuropsychiatric disorders and proceeds following the onset of illness.^5^ Studying the mechanisms that aberrantly regulate the activity-dependent processes facilitating the modeling and strength of synaptic connections in disease may offer insight into the pathophysiology of neuropsychiatric disorders.

One such mechanism is glutamate spillover. Glutamate spillover occurs when synaptically released glutamate diffuses from the synapse and activates extrasynaptic receptors or different active zones in the same synapse.^6,7^ It results in non-specific glutamate binding, activation of glutamate receptors and “runaway” excitatory transmission that may eventually result in neurotoxic cell death.^8,9^

Thus, glutamate clearance is an essential and highly regulated process controlled by a family of transporter proteins that constitute at least 1% of total brain protein.^10^ In this review we will discuss the role that the excitatory amino acid transporters (EAATs) play in the pathophysiology of neuropsychiatric diseases. This extends beyond a diminished ability of transporters to remove glutamate from the synapse, to understanding their multifaceted role in the regulation of synaptic plasticity, a process that is reliant on a complex of interacting proteins that support this essential function.

The nomenclature for EAATs differs between species and has gone through several iterations, particularly for lesser known isoforms. Therefore, all glutamate transporters will be referred to using their EAAT designation throughout the review to ensure clarity. Table 1 describes each variant and provides alternate names.

**Table 1:** Glutamate transporter family nomenclature

Human	Gene	Description	C-terminus sequence	Ensembl/Refseq
EAAT2	SLC1A2	Pan-sequence (C-terminus) 574aa	TLAANGKSADCSVEEEPWKREK	ENST00000278379.7
				NM_004171.3
				NP_004162.2
EAAT2a		Primary sequence (C-terminus) 574aa	TLAANGKSADCSVEEEPWKREK	ENST00000278379.7
				NM_004171.3
				NP_004162.2
EAAT2b		Alternate truncated C-terminus 563aa	HFPFMDIETCI	ENST00000606205.5
				AK298769.1
EAAT2exon9skipping		Exon 9 is not transcribed 529aa	DGGQIVTVLDRMRTSVNVVG	ENST00000278379.7 (EAAT2)
EAAT2exon7skipping		Exon 7 is not transcribed 496aa	LVIMIMWAGTLPVTFRCLEENLG	ENST00000278379.7
				XM_017018139.1
				XP_016873628.1
EAAT1	SLC1A3	Primary sequence 542aa	KKPYQLIAQDNETEKPIDSETKM	ENST00000265113.8
				NM_004172.4
				NP_004163.3
EAAT1exon9skipping		Exon9 not transcribed 497aa	LNFGQIITIRDRLRTTTNVLGDSL	ENST00000381918.3
				NM_001166695.2
				NP_001160167.1
EAAT3	SLC1A1	Primary sequence (C-terminus) 524aa	KSYVNGGFAVDKSDTISFTQTSQF	ENSG00000106688
				NM_004170.5
				NP_004161.4
EAAT4	SLC1A6	Primary sequence (C-terminus) 564aa	YKSLMAQEKGASRGRGGNESAM	ENSG00000105143
				NM_005071.2
				NP_005062.1
EAAT5	SLC1A7	Primary sequence (C-terminus) 560aa	QDEELPAASLNHCTIQISELETNV	ENSG00000162383
				NM_006671.5
				NP_006662.3
Rat				
GLT1	SLC1A2	Primary sequence (C-terminus) 573aa	TLAANGKSADCSVEEEPWKREK	ENSRNOT00000007604.6
				NM_017215.2
				NP_058911.2
GLT1b		Alternate truncated C-terminus 562aa	PFPFLDIETCI	ENSRNOT00000007604.6
				NM_001035233.1
				NP_001030310.1
GLAST	SLC1A3	Primary sequence (C-terminus) 543aa	KPYQLIAQDNEPEKPVADSETKM	ENSRNOG00000016163
				NM_019225.2
				NP_062098.1
GLAST1b		Exon 9 is not transcribed 498aa	QIITIRDRLRT	ENSRNOG00000016163
				NM_001289942.1
				NP_001276871.1
EAAC1	SLC1A1	Primary sequence (C-terminus) 523aa	SYVNGGFSVDKSDTISFTQTSQF	ENSRNOG00000014816.7
				NM_013032.3
				NP_037164.3

The gene name, Ensembl or Refseq identifier and description for human and rat EAATs are listed. Protein sequences for C-terminus (Human: EAAT2a and EAAT2b, EAAT1,3–5; Rat: Glt1, Glt1b and GLAST) or sequences unique to the exon skipping variants (Human: EAAT1exon9 skipping, EAAT2exon7skipping, EAAT2exon9skipping; Rat: GLAST1b) are listed

In this review we will **(I)** outline the basic physiology of glutamate transporters before **(II)** describing recent developments in our understanding of their role in shaping synaptic transmission and plasticity. We will then **(III)** review evidence for dysregulated EAAT expression in disease. Finally, we will summarize the hypothesis that glutamate transporter expression and localization is dysregulated in neuropsychiatric disorders, resulting in aberrant excitatory transmission and synaptic plasticity that contributes to the behavioral phenotypes of schizophrenia and related disorders.

## Basic biology of glutamate transporters

Glutamate is the most abundant excitatory neurotransmitter in the brain. Excessive levels of this amino acid in the synapse results in neuronal death by excitotoxicity.^11,12^ Glutamate is removed from the synapse by active transport mechanisms, an efficient system for terminating glutamate action, thereby maintaining neuronal function.^13^ The EAATs consist of five different membrane bound transporters. EAAT1 and EAAT2 are primarily expressed in glial cells. EAAT2 is responsible for greater than 90% of glutamate transport into crude synaptosomes^14^ and is the predominant glutamate transporter in the brain, except for some regions, including the cerebellum, circumventricular organs and retina, where EAAT1 is the major transporter.^15^ EAAT3-5 are expressed in neurons, with EAAT4 specifically localized to Purkinje cells in the cerebellum and EAAT5 expressed in the retina.^9,16–18^ Cellular localization of EAATs is also diverse with approximately 80% of EAAT2 expression on the cell surface. In contrast, approximately 70% of EAAT3 is expressed in the cytosol.^19^

### Composition of glutamate transporters

**Fig. 1:**
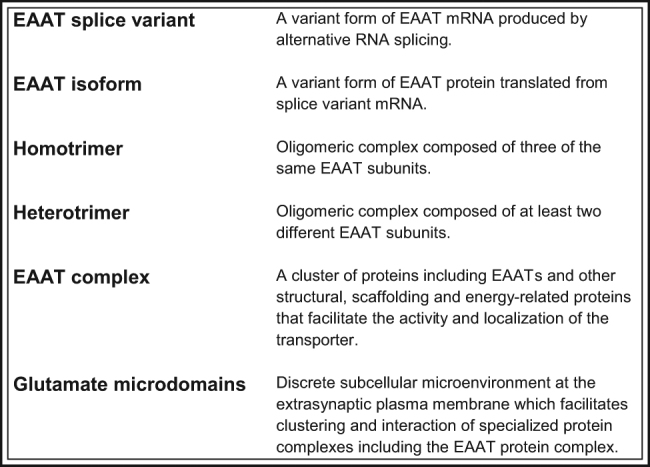
Definitions of terms used throughout review

The glutamate transporter structure was elucidated following determination of the crystal structure of an aspartate transporter from *Pyrococcus horikoshii* ^20^ (Glt_Ph_), which shares 36% amino acid sequence homology with EAAT2.^21^ Residues involved in glutamate binding and transport are highly conserved, up to 90%, between EAATs.^21,22^ EAATs typically form a trimer composed of three identical subunits, called a homotrimer (Fig. 1). Each subunit has eight transmembrane domains (TM1-8) and two hairpin loops (HP1-2), the alternating, symmetrical motion of which allows open and close access to substrate and ion binding sites.^20^ HP1-2 and TM7-8 form the C-terminus, which contains residues important in defining the transport pathway that are highly conserved relative to the N-terminus.^23–25^ TM1-6 supports the transport pathway and mediates interaction between subunits in the trimer.^25^

Yernool et al. (2004) described the multimeric transporter as forming a bowl shape with a solvent-filled extracellular basin that extends halfway across the membrane, supporting an alternating-access model of substrate transport. In an outward facing conformation, the transporter binds to substrate and ions and isomerizes to an inward facing conformation. Following the release of the substrate and ions, the inward facing conformation recycles back to the outward facing conformation.^25^ There is also evidence to suggest that subunits of the EAAT homotrimer act independently.^20,26^ Recently, a study using high-speed atomic force microscopy showed that no more than one domain of Glt_Ph_ is in the inward-facing state at one time, a condition that is ensured by the relatively high energy of the inward state in combination with subunit independence.^27^ Understanding the transporter structure not only informs its function, but is necessary to explain the expression patterns and possible roles of the many different EAAT isoforms.

### EAAT splice variants

There are several post-transcriptionally regulated forms of EAAT, particularly for EAAT1 and EAAT2. Alternative EAAT N-termini and C-termini have been identified, resulting from exon skipping splice variants and alternately spliced 5' untranslated regions and 3' regions.^28–30^ The exon skipping forms include EAAT2 exon7skipping,^31^ EAAT2 exon8skipping,^32,33^ EAAT2 exon9skipping,^34^ EAAT2 exon4skipping,^35^ EAAT2 exon7exon9skipping,^36^ and EAAT2 intron7retaining,^37^ which have all been detected in brain tissue. EAAT1 exon9skipping variant, an EAAT1 splice variant which lacks the region homologous to EAAT2 exon9skipping, has also been described.^38^

EAAT2 exon7- and exon9- skipping variants lack part of the glutamate translocation site. The EAAT2 exon9skipping isoform, identified primarily in white matter astrocytes in rat brain,^39^ may form homotrimers in human brain. Unlike the exon9skipping variant, the exon7skipping isoform is not expressed at the cell surface when expressed alone.^36,40^ EAAT2a (pan-EAAT2 sequence, see Table 1) and EAAT2b (alternate EAAT2 C-terminus, see Table 1) transport glutamate at similar rates when expressed as homotrimers.^41^ EAAT2 exon7skipping or EAAT2 exon9skipping require co-expression with these variants for glutamate transport.^36,40^ High ratios of splice variant EAAT2 exon9skipping to EAAT2a (5:1) significantly reduce glutamate transport. Overall, EAAT exon-skipping variants form heterotrimers (Fig. 1) are expressed on the cell surface, and transport glutamate when expressed with EAAT2a and EAAT2b with differing levels of glutamate transport efficiency.^40^ EAAT1 exon9skipping is expressed in subpopulations of neurons that appear to be dysfunctional, possibly as a result of abnormal local excitation.^42,43^ EAAT1 exon9skipping also lacks glutamate translocation function when expressed alone, but when equal amounts of EAAT1 and EAAT1 exon9skipping are expressed in HEK293 cells, glutamate uptake activity is present, albeit at significantly reduced levels (44% decrease).^38^ Diminished transport may be due to the structure of EAAT1 exon9skipping, which lacks an extracellular domain allowing its transition out of the endoplasmic reticulum (ER). EAAT1 interacts with the splice variant in the ER, reducing the amount of EAAT1 that is expressed at the cell membrane.^38^

### EAAT2b

EAAT2b is the only splice variant, other than EAAT2a, which can transport glutamate as a homotrimeric complex. The EAAT2b unique C-terminus (3'-) sequence was first identified as a variant of EAAT2 in the mouse liver.^29^ The brain variant of EAAT2b, with an N-terminus (5'-) sequence distinct from that of the liver, was subsequently identified by three different groups around the same time.^44–46^ Chen et al. (2002) and Schmitt et al. (2002) published the first complete sequences of what is today considered EAAT2b, using a molecular cloning strategy from a cDNA library prepared from neuronal cultures of transporters with sequence homology to known transporters, and 3'-rapid amplification of cDNA ends and polymerase chain reaction procedures, respectively.^45,46^ An alternately spliced C-terminus replaces the last 22 amino acids with a truncated 11 amino acid long sequence that contains a PDZ (postsynaptic density-95(PSD95)/discs large/zona occludens-1) binding domain^47^ (Table 1). PDZ-binding domains facilitate interactions with other PDZ domain-containing proteins, including multipotent scaffolding proteins that localize clusters of proteins in postsynaptic densities.^48^ For example, EAAT2b interacts with protein interacting with kinase C1 (PICK1), a scaffolding protein that has a regulatory role in AMPA receptor trafficking. This interaction regulates EAAT2 via protein kinase C (PKC), suppressing EAAT2 downregulation following PKC activation.^47^ PICK1 increases EAAT2b mediated leak current, also impacting glutamatergic neurotransmission.^49^ Finally, EAAT2b also interacts with PSD95, allowing for transporter clustering at the postsynaptic density, thus increasing transporter activity.^50^

The EAAT2b-unique PDZ-binding domain provides a mechanism by which EAAT2b trafficking to the plasma membrane is modulated. Unlike EAAT2a, which is constitutively cycled to the membrane, Underhill et al. (2015) have shown that stable EAAT2b localization at the cell surface is achieved by its interaction with disks large homolog-1 (DLG1), a PDZ domain protein. AMPA-mediated increase in intracellular calcium levels leads to the activation of Ca^2+^/calmodulin-dependent protein kinase II, resulting in phosphorylation of DLG1 and its dissociation from EAAT2b. This destabilizes EAAT2b localization at the cell surface.^51^ Therefore, while EAAT2a constitutively cycles to the cell surface, EAAT2b is trafficked and expressed at the plasma membrane in response to extracellular signaling. This allows for consistent basal glutamate uptake and provides a mechanism to alter uptake in response to signaling via modulation of EAAT2b cell surface expression. In EAAT2 heterotrimers containing both EAAT2a and EAAT2b, there is evidence that EAAT2a may indirectly interact with PDZ domain proteins via EAAT2b, although the functional consequences of this observation are unknown.^50,52^ Overall, the role of EAAT splice variants and particularly those expressed in neurons is not well understood.

### Neuronal EAAT2 expression: function and relevance

Neuronal expression of EAAT2, long considered primarily an astrocytic transporter, is now well-established after many years of research, as comprehensively discussed by others.^48,53,54^ EAAT2 expression in neurons appears to be primarily presynaptic, in axon-terminals, whereas the neuronal transporter EAAT3 is expressed somato-dendritically. Originally, EAAT2b was a candidate for the neuronally expressed transporter isoform.^53^ It is now known that EAAT2b is primarily expressed in astrocytes in normal brain, while EAAT2a is found in both astrocytes and neurons.^55–58^

### Function of neuronal EAAT2

Although less than 10% of EAAT2 is expressed neuronally,^57^ it appears to function in a similar manner to astroglial EAAT2 and maintains its role in glutamate clearance.^53,54,59^ Glutamate reuptake into synaptosomes is a technique to directly assess EAAT activity. However, for many years, most glutamate reuptake in the brain was considered a function of astrocytic EAAT2, ignoring evidence describing glutamate uptake into nerve terminals.^15^ Furness et al. (2008) confirmed disproportionately high reuptake of glutamate into crude synaptosomes and hippocampal slices (approximately 50% reuptake), relative to the amount of EAAT2 expressed in neurons (less than 10% total EAAT2 protein expression). This discrepancy was probed in a study using a model of astrocyte-specific or neuron-specific knockout of EAAT2.^60^ Knockout of EAAT2 in astrocytes resulted in an almost complete loss of EAAT2 protein expression, but a nonsignificant reduction of glutamate uptake into synaptosomes. Glutamate reuptake into synaptosomes from mice with neuronal knockout of EAAT2 was significantly reduced (approximately 40%), although neuronal EAAT2 protein is only 6% of total EAAT2 expression.^60^ This study also measured glutamate reuptake from forebrains of astrocyte or neuron EAAT2 knockout mice reconstituted into liposomes, to account for inefficient astrocyte membrane resealing during synaptosome preparation. Astrocyte-specific knockout of EAAT2 resulted in glutamate reuptake into liposomes that was reduced by approximately 75%, a value that correlated with the EAAT2 protein levels measured by immunoblot. This indicated that astrocyte reuptake was not fully accounted for in crude synaptosomes, due to limitations of the preparation method. There was no significant effect of neuronal EAAT2 knockout on glutamate uptake in liposomes, as expected due to the relatively low levels of neuronal EAAT2 protein in this preparation. As suggested by Petr et al. (2015), neuronal EAAT2 expression likely contributes to glutamate clearance, however a combination of synaptosome and liposome preparations is required to elucidate the relative contribution of astrocyte and neuronal EAAT2 to glutamate uptake.

Presynaptic expression of EAAT2 may act to replenish stores of presynaptic glutamate, bypassing the glutamate-glutamine cycle. Typically, glutamate was thought to be primarily recovered by EAAT2 expressed in astrocytes. Within astrocytes, glutamate is converted to glutamine, transported to presynaptic neurons, converted back to glutamate, packaged in vesicles, and released as a neurotransmitter. The relative importance of direct presynaptic reuptake versus the traditional glutamate–glutamine cycle in restoring presynaptic glutamate has been debated,^59,61,62^ although the primary role of glutamate transport remains the modulation of excitatory transmission.^63,64^

EAAT2 is expressed in many types of nerve terminals^53^ but not in all neurons, including in the Calyx of Held where electrophysiological studies failed to find evidence of EAAT currents.^57,60,65^ As a result of the inaccessibility of presynaptic terminals to electrophysiological recording, most studies investigating the response of EAATs to synaptically released glutamate are conducted in cells with large glutamatergic terminals, including retinal bipolar cells. Thus, study of retinal cells might provide novel insight into the presynaptic role of EAATs expressed in other brain regions.

EAAT5 is localized to presynaptic retinal cells although there are reports of EAAT2 expression in these cells as well.^66–69^ EAAT5 transport rates are slow, but EAAT5 gates large Cl^−^ currents,^70^ suggesting a role as an inhibitory receptor. Dysregulation of EAAT-mediated anion selective conductance may play a role in neurological disorders.^71^ EAAT5-associated anion conductance regulates synaptic transmission by fast hyperpolarization of the axon terminals. Released glutamate activates presynaptic EAAT5, initiating a negative feedback control on transmitter release via activation of transporter-associated anion currents.^72,73^ In rod to bipolar cell synapses, EAATs located presynaptically on rod cells are also responsible for glutamate clearance, with glial Muller cells and postsynaptically located EAATs playing a minimal role.^74^ This requires a high density of EAAT expression on the terminal for efficient clearing and may also enable signal transmission without crosstalk, as glutamate diffusion is constrained to the synaptic cleft. EAAT5 is the primary retinal transporter and it is unknown what, if any, impact retinal EAAT2 may have had in these studies. However a similar mechanism of presynaptic regulation may extend to neuronal EAAT activity in other cell types in the brain. The structural conformation of EAAT2 limits its role as a chloride channel, but as discussed by Sery et al. (2015), small structural changes in a yet to be identified EAAT2 variant could permit such a molecule to become permeable to chloride and act in an inhibitory manner, inducing presynaptic feedback inhibition.^63^ The specific role(s) of presynaptically localized EAAT2 isoforms are yet to be elucidated,^53,54^ but are of much interest as aberrant expression of neuronal EAAT2 is implicated in the pathophysiology of neuropsychiatric disorders, as discussed below.

### Glutamate transport

Glutamate transport is coupled to the cotransport of three Na^+^ ions and one H^+^ ion with the counter transport of one K^+^ ion and is linked to an uncoupled Cl^−^ anion current.^75,76^ Glutamate binds to EAAT transporters at rates of 10^6^–10^7^ M^−1^ s^−1^,^77^^–^^79^ with a capture efficiency of 0.5 for EAATs 1–3.^77,80^ This means that glutamate is rapidly bound to transporters and is either released or, at equal rates, transported into the cell. Unbound glutamate quickly encounters other available transporters, given the high density of EAATs that are found near the synapse, and will once again be sequestered or transported, preventing glutamate spillover from the synapse. This combination of buffering, transport and high density expression of transporter explain how EAATs clear glutamate in response to fast synaptic events, even though they have relatively slow cycling and high glutamate saturation rates. Other factors that influence uptake, including differences in EAAT subtype and regulation of the cell-surface localization of transporters near synapses are not as well understood.^9^ Disruption of this process can result in glutamate spillover.^81,82^

### Metabolic complexes and the glutamate transporter

Glutamate transport is an energy dependent process and requires substantial metabolic support.^13,83^ EAAT2 forms a protein complex of structural and metabolic proteins, linked to mitochondria, which support the high-energy process of glutamate reuptake.^84^ Compartmentalizing these proteins within a microdomain allows for the spatial coordination of glutamate transport with the energy needs of the cell.^85^ In addition, the variable extracellular concentration of glutamate, thought to be in the nanomolar range in the synaptic cleft and micromolar range in extrasynaptic spaces, suggests a role for discrete glutamate microdomains.^86–88^ The localization of glutamate receptors and transporters outside synapses suggests partitioning of extrasynaptic spaces in which glutamate concentrations are independently maintained,^89^ although a recent study suggests that extrasynaptic glutamate levels are constant across extracellular compartments, a finding inconsistent with localized microdomains with locally higher glutamate levels.^90^ EAAT1 also colocalizes with mitochondria and glycolytic enzymes,^91,92^ although EAAT1 and EAAT2 do not interact and are part of different protein complexes.^93^

Na^+^/K^+^-ATPase, a transmembrane enzyme with multiple isoforms consisting of different α and β subunits that hydrolyze ATP,^94^ colocalizes with glutamate transporters in astrocytic processes.^95^ Na^+^/K^+^-ATPase generates an electrochemical gradient of high levels of K^+^ and low Na^+^ internally, providing the energy for glutamate uptake, which cotransports one glutamate molecule and three Na^+^ into the cell and one K^+^ out of the cell.^15,96,97^ In addition, EAAT2 interacts with different isoforms of Na^+^/K^+^-ATPase, providing a putative mechanism to modulate the localization and activity of these transporter complexes, as both glutamate transporters and Na^+^/K^+^-ATPase regulate the other’s membrane trafficking and localization, providing localized control of glutamate uptake.^84,98^ The main source of energy for the Na^+^/K^+^-ATPase-glutamate transport complex is ATP, generated by glycolysis.^99,100^ Hexokinase facilitates the first committed step in the metabolism of glucose, and colocalizes with mitochondria to the EAAT2 protein complex.^85,101^ Juxtaposition of mitochondria with EAAT2 in microdomains in astrocyte processes is increased in response to neuronal activity.^102^ Conversely, mitochondria are immobilized in processes in response to EAAT activation by neuronal release of glutamate. Thus, mitochondria are mobilized and retained when EAAT2 clusters near the synapse.^102,103^ The network of proteins that support the energy needs of the EAATs are necessary to not only clear glutamate from the synapse but support their role in shaping synaptic plasticity.

## Complex biology of glutamate transporters: ability to modulate synaptic plasticity

Schizophrenia and other neuropsychiatric illnesses are considered disorders of neuroplasticity.^104^ Can pathological dysregulation of EAAT2 contribute to the synaptic plasticity-related symptoms of disease? EAAT2-mediated clearance of glutamate is a dynamic process that integrates glial-neuronal communication to fine-tune the spatial and temporal actions of the glutamate transporter in order to modulate excitatory transmission and synaptic plasticity. Regulation of glutamate reuptake plays a role in memory formation in vertebrates and invertebrates, and may be a common mechanism of plasticity at glutamate synapses.^105–108^ Studies in Aplysia showed that neuronal glutamate reuptake modulated the time course of excitatory post synaptic potentials in sensory neurons of pleural ganglia, playing an important role in synaptic transmission. A role for glial transporters could not be ruled out in this study.^109^ In rodent hippocampus, the authors reported increased neuronal glutamate reuptake in fear conditioning and early-long-term potentiation (LTP; EAAT3) and late-LTP (EAAT2), indicating differing roles for transporters at different subcellular locations.^106,109,110^ EAAT3 and EAAT2 levels were also increased following activation of different signaling pathways during early and late LTP, resulting in phosphorylation of EAAT3 and EAAT2, by PKC and PKA, respectively.^110^ These studies provide insight into the potential complexity of glutamate transporter regulation in synaptic plasticity. Recently, several studies have directly addressed the role that EAAT2 glutamate clearance has on neuroplasticity.

### Neuronal activity controls transporter localization

EAATs form distinct clusters in astrocyte membranes located near synapses, with estimates that 82% of transporter clusters are found within 1 µm of the synapse.^111^ The density of EAAT2 clusters and their perisynaptic localization increases or decreases in response to pharmacological stimulation or blockade, respectively, of neuronal activity, suggesting that alterations in neuronal activity result in altered transporter expression.^111,112^ In addition, the rate at which transporters cluster may have a significant functional effect on synaptic plasticity, as demonstrated by dysregulated activation of extrasynaptic receptors if astrocytic transporter localization trails behind synapse development.^113^

The glial response of altering the number and density of transporters in response to changes in neuronal activity might be a mechanism to maintain glutamate homeostasis. Homeostatic synaptic plasticity is a negative feedback mechanism of regulating the rapid, input-specific changes associated with activity-dependent (Hebbian) plasticity.^114–116^ In instances of increased neuronal activity, it has been proposed that neurons compensate by decreasing neurotransmitter release (presynaptically) or by trafficking receptors from synaptic to extrasynaptic sites (postsynaptically).^114^ This maintains the relative strength of synapses in response to a global change in activity.^117^ EAAT mobility and regulation via presynaptic signaling may act as a mechanism to maintain homeostasis.

### Temporal effect on pre-synaptic and post-synaptic activity

Using a model of Hebbian plasticity, spike-timing-dependent plasticity (STDP), Valtcheva and Venance (2016) demonstrate the instrumental role of EAAT2 in synaptic plasticity. LTP and long-term depression (LTD) are the most well-known forms of Hebbian plasticity and allow for the study of the molecular processes underlying memory.^118–120^ STDP is typically bidirectional, highly sensitive to timing, and dependent on paired pre-and post-synaptic activity. LTP results from consistent firing of presynaptic spikes 0–20 ms before postsynaptic cells. In STDP, LTD occurs when the firing order is reversed or there is loss of correlation of pre-and post-synaptic firing order at approximately 100 ms.^121,122^ STDP is commonly found at excitatory synapses throughout the brain. Blocking EAAT2 resulted in the replacement of STDP with a timing-independent, non-Hebbian form of plasticity that also did not require the usual order of activity (i.e., pre-synaptic before post-synaptic spikes). In addition, increasing EAAT2 expression by administering ceftriaxone prevented STDP and the emergence of plasticity.^123^ Thus, EAAT2 activity gates the transition from non-Hebbian to Hebbian plasticity, temporally regulating pre-synaptic and post-synaptic glutamate activity.^123^

### EAAT2 localization and synaptic activity

In addition to a temporal role in modulating synaptic transmission, synaptic plasticity is dependent on EAAT2 localization. EAAT2 immobilization, by antibody crosslinking, attenuated transporter surface diffusion and significantly impacted rise and decay times of excitatory post synaptic currents (EPSCs), suggesting altered glutamate clearance from the synapse.^81^ Immobilization had no effect on total EAAT2 reuptake, suggesting that effects on synaptic transmission were due to attenuated lateral diffusion of the transporter. As discussed earlier, glutamate transport is a relatively slow process relative to the duration of elevated glutamate in the synaptic cleft and so requires availability of a large number of transporters at the membrane which can buffer glutamate.^124^ Surface mobility is required for EAAT2 to diffuse into the synapse, bind glutamate and rapidly diffuse away from the synapse so that non-bound transporters can move into position to buffer or clear glutamate. EAAT2 surface diffusion is increased on astrocytic processes that surround activated synapses.^125^ The rapid movement of glutamate-bound transporters from sites near activated synapses may allow for positioning of unbound transporters and efficient buffering of glutamate.^81,125^ An increase in EAAT2 surface mobility may also reduce glutamate spillover.^126^ The mechanism by which the rapid dispersal of EAAT2 is regulated in response to increased activity has yet to be elucidated. It has been proposed that uptake of glutamate changes the conformation of EAAT2, resulting in dissociation of the transporter from scaffolding proteins and increasing its surface mobility.^125^ EAAT2b, likely due to the PDZ domain which allows it to bind structural proteins like PSD95, PICK1 and DLG1,^47,50,51^ is more confined to astrocyte domains near synapses than the non-PDZ domain containing EAAT2a isoform.^125^ EAAT2a-EAAT2b heterotrimers might also be stabilized at the membrane through EAAT2b-protein interactions. Altered phosphorylation may also play a role in EAAT2 regulation, as reviewed extensively elsewhere.^127–131^ Further study is required to determine what role scaffolding proteins play in facilitating rapid surface diffusion of EAAT2 in response to increased glutamate.

In summary, EAAT2 is sensitive to both neuronal and astrocytic activity, facilitating EAAT2’s role in maintaining glutamate homeostasis. EAAT2 surface diffusion increases in response to high levels of activity and decreases in response to low activity levels. Thus, EAAT2 mobility likely regulates the concentration of glutamate at the synapse, directly affecting synaptic transmission and synaptic plasticity.

### EAAT2 modulates synaptic transmission via astrocyte morphology

EAAT localization near the active zone of the synapse is required to modulate glutamate levels and set the magnitude of synaptic transmission.^132^ Connexin30 is a gap junction protein that induces structural changes in the brain.^133^ In a connexin30 knockout model, astrocyte morphology was altered, resulting in dysregulation of the extent of astrocyte processes into the synaptic cleft.^132^ Excitatory neurotransmission was decreased in these animals, driven by altered glutamate clearance via EAAT2. Reduced levels of synaptic glutamate also affected excitatory synaptic strength and reduced LTP. Astrocyte processes are plastic and allow for dynamic, activity-dependent organization of EAAT2 at the synaptic cleft. The spatial localization of EAAT2 on astrocyte processes is essential for regulating synaptic glutamate levels.

### Regulation of EAAT2 modulated plasticity

The mechanisms that spatially and temporally regulate EAAT localization are not fully understood, although a number of studies have addressed this question. Oliet et al. (2001) found that transmitter release is modulated presynaptically in response to reduced glial coverage of neurons and reduced clearance of glutamate by EAAT2. The resulting increase in synaptic glutamate levels led to activation of G_i_-coupled presynaptic group III metabotropic glutamate receptors (mGluRs), which in turn reduced presynaptic glutamate release.^134,135^ A recent study addresses how neuronal activity regulates glutamate reuptake in response to high-frequency stimulation. Following presynaptic activity bursts of greater than 30 Hz, glutamate uptake is slowed by up to three-fold. Extending the time glutamate persists in the synapse yielded dynamic local modulation of synaptic activity following presynaptic activity.^136^ Other work has focused on the depolarization state of astrocytes. For example, Armbruster et al. (2016) propose that presynaptic activity reduces EAAT2 activity by initiating localized depolarization of astrocyte microdomains. Neuronal activity can depolarize astrocytes,^137^ and glutamate induced Ca^2+^ signals are restricted to microdomains in glial processes.^138^ There is also some evidence that astrocyte membrane potential can be compartmentalized.^136,139,140^ Supporting this hypothesis is the putative role of astrocytic microdomain complexes providing metabolic support to power the Na^+^/K^+^ATPase-driven EAAT2 glutamate clearance. These hypothetical compartments likely contain a network of EAAT2-interacting proteins, including structural and anchoring proteins that could fulfill other functionally-dependent synaptic roles.^85,141,142^ Their localization in proximity to synapses suggests that EAAT2 microdomains regulate transporters on astrocytes.^81,136^ It is unclear if EAAT2 microdomains are present on presynaptic terminals. Presynaptic neurons indirectly regulate astrocytic EAAT2 expression, via binding of the neuron-stimulated nuclear ribonucleoprotein K transcription activator to the glial EAAT2 promoter.^143^ Neuron-induced epigenetic changes in the EAAT2 promoter are associated with neuron-dependent EAAT2 mRNA increase in astrocytes.^144^ EAAT2 levels are also indirectly regulated by microRNAs (miR), small, noncoding RNAs. Exosomes containing miR-124a-containing exosomes, isolated from neurons and internalized by astrocytes, result in upregulation of EAAT2 protein levels but not mRNA.^145^ Presynaptically expressed miR-137 regulates the vesicular glutamate transporter which loads synaptic vesicles with glutamate,^146^ and results in downregulation of target genes including synaptotagmin, impairing the release of synaptic vesicles.^147^ Postsynaptically, miR-137 is upregulated in response to mGluR5 activation and directly regulates AMPA-type glutamate receptors.^148^ Overexpression of miR-137 results in reduced AMPA-mediated transmission,^148^ suggesting a key role for miR regulation in synaptic plasticity at the excitatory synapse.

In summary, there is evidence of direct modulation of synaptic plasticity by EAAT2, a process that is at least partially modulated by presynaptic neuronal activity. The mechanism of presynaptic modulation of EAAT expression remains to be elucidated, but may include miR or other processes. These findings highlight the importance of EAAT2 localization and trafficking for normal synaptic function, and provide a theoretical basis for novel pathophysiological mechanisms in neuropsychiatric diseases.

## Glutamate transporters in neuropathological conditions

**Fig. 2:**
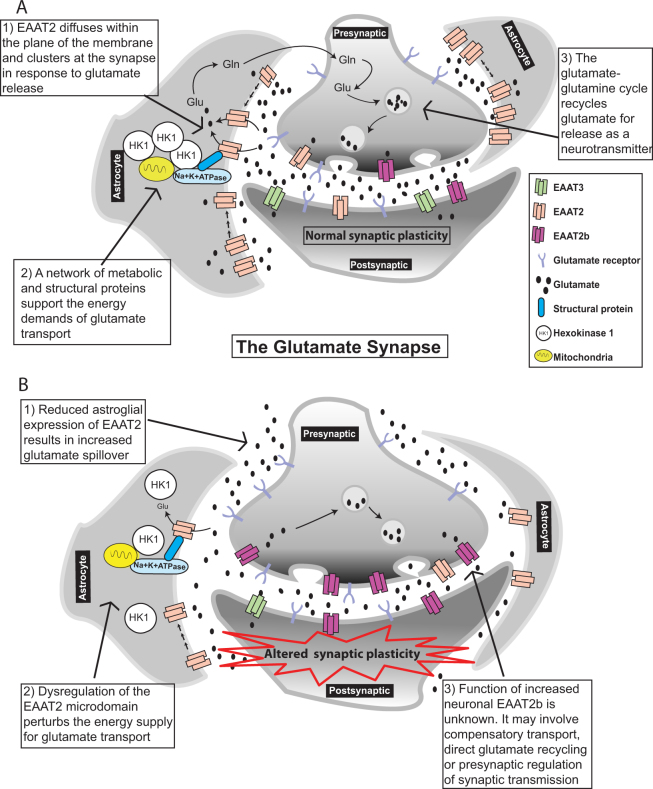
**a** Glutamate transporter action and regulation in the glutamate synapse. Astrocyte processes expressing EAAT2 extend into, and around, the synapse. Glutamate is primarily cleared from the synapse by astroglial localized EAAT2. EAAT3, located postsynaptically, and EAAT2(b), located pre- and postsynaptically, are responsible for less than 10% of glutamate reuptake (1). A microdomain of proteins including EAAT2 and mitochondria supports the active transport of glutamate (2). Following transport, glutamate (Glu) is converted into glutamine (Gln) in the astrocyte for transport to the neuron where it is metabolized back to glutamate and packaged into vesicles for release as a neurotransmitter (3). **b** Glutamate transporter action and regulation in the glutamate synapse in schizophrenia: a role for EAAT2b. EAAT2b levels are significantly increased in neurons in schizophrenia. Although EAAT2b homotrimers transport glutamate, increased expression of EAAT2b is not sufficient to compensate for the general loss of glial EAAT2 activity in schizophrenia. Changes in localization of EAAT2b affect glutamate reuptake and contribute to spillover (1). The EAAT2 microdomain is disrupted in schizophrenia. Hexokinase 1 (HK1) is dissociated from mitochondria, implying altered metabolism. Reduced ATP levels impact Na^+^/K^+^ ATPase efficiency and glutamate reuptake (2). The extension of astrocyte processes, and therefore EAAT2 localization at the synapse, significantly impacts glutamate reuptake and synaptic plasticity. Presynaptically expressed transporter, for example EAAT5, regulates synaptic transmission by initiating a negative feedback control of glutamate release. It is not yet known if EAAT2b or another EAAT2 variant can directly regulate excitatory transmission by such a mechanism. Neuronal EAAT2b recycling of glutamate bypasses the glutamate–glutamine cycle. However, the exact function of neuronal EAAT2b expression in disease has yet to be elucidated (3). EAAT2b localization, expression, metabolic support and regulation is significantly altered in disease. This results in aberrant excitatory transmission and synaptic plasticity which contribute to the pathophysiology of neuropsychiatric illnesses

We propose that dysregulation of EAAT expression, as demonstrated by its altered ultrastructural and cellular localization and disruption of its glial microdomain, regulates excitatory transmission and shapes synaptic plasticity. This may result in altered neuronal signaling and circuit-wide plasticity deficiencies that could contribute to the cognitive and other symptoms associated with schizophrenia and neuropsychiatric illnesses (Fig. 2).

### Spillover as mechanism of disease

Glutamate spillover may be a pathophysiological feature of many neuropsychiatric disorders including schizophrenia, depression and addiction. An increase in spillover can result in non-specific activation of glutamate receptors and activation of downstream signaling mechanisms. Spillover of glutamate is sufficient to induce plasticity, in the form of LTP, in heterosynaptic pathways in fear conditioning, whereas glutamate reuptake is essential to maintain specificity of synaptic modification in circuitry of fear learning.^149^ Reuptake also offers control of spatial specificity of long-term modifications at the synapse, as it places temporal limits on the action of glutamate at synaptic receptors. However, physiologic spillover is necessary for signal transmission and activation of receptors on adjacent synapses in some brain regions including the hippocampus and cerebellum.^9,13,150,151^ It may be that a mechanism that is physiological in some areas may be pathological in other brain regions or circuits.

A role for dysregulation of the glutamate transporter has been established in the neurobiology of addiction. Glutamatergic circuitry is essential for extinction learning and recall of extinction memory following cocaine exposure.^152^ Glutamatergic projections to the nucleus accumbens from the frontal cortex, amongst other regions, are necessary for cue-induced relapse to drug seeking behavior. Chronic drug exposure alters synaptic plasticity at glutamate synapses in the nucleus accumbens, leading to the enduring symptoms of addiction and vulnerability to relapse.^152,153^ Decreasing glutamate reuptake, by reducing EAAT2 levels, disrupts extrasynaptic glutamate levels and alters synaptic plasticity, possibly via altered activation of mGlu receptors.^153,154^ Taken together, these mechanisms may underlie some drug seeking behaviors.

The mechanism by which EAAT2 and impaired glutamate homeostasis contribute to drug-seeking behavior was investigated.^153^ In a model of addiction, Shen et al. (2014) demonstrated the central role of synaptic glutamate spillover.^82^ EAAT2 levels are reduced in heroin-dependent rats in a model of heroin reinstatement. Using changes in *N*-methyl-D-aspartate (NMDA) receptor EPSC decay time as a measure of synaptic spillover, the authors demonstrated an EAAT2-dependent increase in decay time in heroin-treated rats compared to controls. These data show that reduced EAAT2 levels resulted in spillover of glutamate that contributed to the pathophysiology of addiction behavior.^82^ Interestingly, ceftriaxone, a drug that increases EAAT2 expression, attenuated reinstatement of heroin-seeking behavior.

EAAT2 upregulation was also essential for ceftriaxone attenuated reinstatement of cocaine-seeking behavior in the nucleus accumbens, an effect that was prevented by EAAT2 knockdown.^155^ Ceftriaxone is a cephalosporin antibody of the β-lactam family that enhances EAAT2 expression and function when administered chronically.^153^ Increased glutamate reuptake in normal animals administered with ceftriaxone resulted in reduced presynaptic mGlu receptor activation and reduced kainate receptor activation.^156^ These changes may impact synaptic plasticity not only by increasing transmitter clearance, preventing glutamate spillover, but also by reducing activation of presynaptic autoreceptors that regulate the release of glutamate.^156^ In addition to its effect on presynaptic receptor activation, ceftriaxone also prevented cocaine reinstatement by reducing medium spiny nerve stimulation by postsynaptic mGluR5 and AMPA receptors in the nucleus accumbens, following EAAT2 upregulation.^155^ Ceftriaxone did not regulate EAAT2 via a transcriptional mechanism or by interaction with the cysteine–glutamate exchanger in this study. While the precise mechanism of increased EAAT2 is unknown, the use of ceftriaxone has revealed novel pathological processes in animal models of addiction. We propose that glutamate spillover via alterations in EAAT2 expression and localization may be a pathophysiological mechanism in neuropsychiatric diseases. We review evidence supporting this hypothesis below.

### Altered expression of EAAT2 in severe mental illness

The glutamate hypothesis of schizophrenia is based on observations that administration of the NMDA receptor antagonist phencyclidine (PCP) induces schizophrenia-like symptoms, including psychosis and cognitive impairment.^157,158^ This hypothesis encompasses the cognitive, negative and positive symptoms of schizophrenia and offers promising new substrates for pharmacological intervention. The glutamate hypothesis and its importance in schizophrenia are rigorously reviewed elsewhere.^159^

PCP impairs prepulse inhibition (PPI) of the startle reflex, a simple form of information processing that is consistently reduced in schizophrenia.^160^ PPI, a well-established endophenotype of schizophrenia,^161^ is a measure of dysfunctional social processing, a deficit that is intermediate to part of the underlying molecular causes and the clinical symptoms of disease.^162^ The Research Domain Criteria (RDoC) initiative recognizes PPI as a means to assess perception, a key construct of the cognitive systems domain, and the subconstruct auditory perception, as outlined in the RDoC matrix. Accordingly, evidence suggests that glutamate transport impacts this construct. For example, ceftriaxone-mediated increase in EAAT2 expression resulted in impaired PPI in a rodent model.^163^ Rats treated with a combination of ceftriaxone and acute PCP had greater impairment in PPI than either treatment alone, suggesting that a combination of NMDA antagonism and EAAT2 upregulation operate through non-overlapping mechanisms to negatively affect glutamate transmission.^164^ Interestingly, chronic, but not acute, PCP administration increases EAAT2 expression, reducing extracellular glutamate concentrations.^165^ Acute PCP acts through a different mechanism, reducing expression of presynaptic proteins involved in glutamate transmitter release to reduce extracellular glutamate, with no significant effect on EAAT2 reuptake.^166^ As discussed above, ceftriaxone-induced increase in EAAT2 expression are likely related to presynaptic mGluR activation.^156^ Administration of an mGluR2/3 agonist prevented the PPI impairment associated with ceftriaxone-induced EAAT2 upregulation.^167^ The likely mechanism by which overexpression of EAAT2 causes PPI impairment is via glutamate spillover, which mGluR2/3 relies on for activation due to its perisynaptic localization.^168^ Clozapine, an antipsychotic used in the treatment of schizophrenia, specifically downregulates astrocytic EAAT2 levels when administered orally at 25–35 mg/kg/day for three weeks, resulting in increased extracellular glutamate levels.^169,170^ Clozapine increased the expression of the presynaptic protein synaptophysin, which is involved in neurotransmitter release, in the same regions where EAAT2 was decreased. This suggests an increased potential for glutamate release that may facilitate the role of clozapine in potentiation of the excitatory synapse.^171^ Overall, pharmacological (PCP) and behavioral (PPI) paradigms that are associated with the symptoms of schizophrenia implicate not only glutamate receptor dysregulation in schizophrenia, but other proteins related to glutamate transmission, including EAAT2.

### Metabolic complexes and glutamate transporter microdomains in disease states

In disease states, two studies found evidence that the integrity of the EAAT2 protein complex is disrupted.^142,172^ In human prefrontal cortex, EAAT2 protein complex interactions include Na^+^/K^+^ATPase, hexokinase 1 and aconitase.^142^ In fractionation studies, expression levels of hexokinase 1 were significantly increased in extrasynaptic fractions (localized to extrasynaptic membranes and cytosol) compared to mitochondrial fractions in schizophrenia subjects.^142^ Reduced hexokinase 1 levels in the mitochondrial fraction suggest dissociation of hexokinase from mitochondria, as hexokinase is normally associated with mitochondria in the brain.^173^ Hexokinase 1 attachment to the outer membrane of mitochondria was also reduced in the parietal cortex in schizophrenia.^172^ It has been speculated that disruption of the association of hexokinase with mitochondria may decrease ATP production and could have implications for Na^+^/K^+^ATPase efficiency, possibly resulting in impaired glutamate reuptake.^142,173^ Alternatively, alterations of hexokinase 1 localization in schizophrenia may reflect changes in the localization of EAAT2 at perisynaptic sites.^142^ Supporting this hypothesis, G-protein pathway suppressor-1 protein is significantly upregulated in the frontal cortex in schizophrenia. This protein binds EAAT2, regulating its surface trafficking via a leucine zipper-like motif.^174^ In addition, glycosylation of EAAT2 is significantly reduced in schizophrenia, a posttranslational modification that is involved in molecular trafficking.^175,176^ Interestingly, increased levels of EAAT2b, but not EAAT2a or EAAT2 exon9skipping protein isoforms, were identified in the extrasynaptic membrane fraction in schizophrenia.^142^

Overall, these data suggest abnormal subcellular partitioning of EAAT microdomain proteins in a disease with prominent cognitive deficits. Altered energetic and structural support will affect EAAT2 diffusion and localization near the synapse, a process that is required to maintain excitatory transmission and modulate synaptic plasticity.^9^ A yet to be confirmed role for EAAT2 microdomains in regulating transporter surface diffusion has been proposed, a process that might be disrupted in schizophrenia and other disorders.^89^ Further, presynaptic regulation of EAAT2 clearance in astroglia may also rely on the fidelity of astrocytic microdomains.^136^ Since EAAT2 plays a central role in synaptic plasticity and neurotransmission, disruption of the metabolic and structural complexes in microdomains that enable EAAT2 clearance of glutamate suggests a role for atypical transporter localization in the pathophysiology of brain diseases.

### Further evidence of altered EAAT2 expression in postmortem brain

Postmortem studies have identified altered expression of EAAT2 in several pathological conditions other than schizophrenia. Altered expression of glutamate transporters in experimental models are comprehensively reviewed elsewhere.^177–179^ The following is an overview of the dysregulation of primarily EAAT2 and related splice variants in human disease.

### Amyotrophic lateral sclerosis (ALS)

EAAT2 protein is significantly reduced in ALS in the motor cortex and spinal cord^180–182^ with evidence of increased glutamate concentration in cerebrospinal fluid^183^ and reduced uptake in the brain.^184^ Recently, increased EAAT2 pre-mRNA editing has been reported in the spinal cord and motor cortex, but not cerebellum, of ALS subjects, suggesting premature termination of transcription and reduced levels of EAAT2 in disease-relevant regions.^185^ Cleavage of EAAT2, by caspase-3 at a consensus site in the C-terminus, results in inhibition of the transporter and may contribute to excitotoxicity induced loss of motor neurons in ALS.^186^

EAAT2b protein is increased in ALS and was found in pyramidal neurons rather than its typical localization to astrocytes.^184,187^ The role of neuronal EAAT2b is unclear. Although upregulation of EAAT2b could be considered a compensatory mechanism for reduced EAAT2a levels, increased expression of this isoform alone is unable to maintain physiological clearance of glutamate at the synapse.^177,187^

### Alzheimer’s disease

Significantly lower levels of EAAT2 gene expression were reported in Alzheimer’s disease subjects in the cortex.^36,188–190^ In Alzheimer’s disease, EAAT2b immunoreactivity was decreased in the motor cortex.^188^ In the cerebral cortex, the levels of EAAT2b mRNA were reduced but the proportion of EAAT2b: EAAT2a expression was unchanged relative to disease severity.^36^ In the same study, gene expression of EAAT2 exon7skipping and EAAT2 exon9skipping were significantly increased relative to EAAT2a expression and disease severity. This suggests that the regulation of EAAT splice variants is independent of EAAT2a regulation.^36^ Although splice variants make up a small proportion of total EAAT2, an imbalance in splice variant expression may result in less stable EAAT2 heterotrimers.^40^ Under pathological conditions, even small changes in the expression of EAAT2 isoforms localized to sites of vulnerability or stress can impact glutamate transport.^191^ For example, an increase in the proportion of exon-skipping variants relative to EAAT2a may reduce glutamate reuptake without impacting total EAAT2 expression. The correlation of EAAT expression with disease severity, in combination with the increased splice variant expression that can facilitate in reduced glutamate reuptake, suggests increased glutamate levels in vulnerable brain regions in Alzheimer’s disease.^36^

The cell-specific expression of EAAT variants in Alzheimer’s disease may provide insights into this devastating illness. Pow and Cook (2009) identified exon-skipping isoforms of EAATs in neurons in temporal cortex in Alzheimer’s disease. Exon3skipping isoforms of EAAT1 and EAAT2 and exon9skipping isoforms of EAAT1, 2 and 3 were localized to cortical neurons in disease with low levels or no expression in astrocytes or control tissue.^191^ The altered expression of EAAT variants in neuronal cells under pathological conditions suggests that changes in EAAT localization may play a neuron-specific role in disease. Although the consequences of this shift in expression are not fully understood, such changes in transporter expression may contribute to the aberrant synaptic plasticity found in Alzheimer’s disease.

### Schizophrenia

In addition to the data discussed above on metabolic complexes, several studies have examined EAAT2 expression in schizophrenia. Reports of EAAT expression in schizophrenia vary based on the region and substrate being measured.^179^ For example, EAAT2 mRNA, protein and activity were increased in the prefrontal cortex (Brodmann area 9 and 10),^192^ but others reported decreased mRNA,^193^ or no change in mRNA or protein in the dorsolateral prefrontal cortex (DLPFC) and anterior cingulate cortex (ACC).^174^ In other brain regions examined, there appears to be an overall reduction in EAAT expression. EAAT2 protein expression was decreased in the hippocampus, superior temporal gyrus and Brodmann area 10.^194^ Reduced glycosylation, a post translational modification important for EAAT2 localization to the membrane, was found in the DLPFC.^176^ EAAT2 protein was also significantly reduced in the mediodorsal and ventral tier nuclei in the thalamus following immunoblotting. EAAT1 protein was reduced in the mediodorsal nucleus only.^195^

Region-level studies may mask subtle changes in EAAT expression in different cell populations, indicating a need for cell-specific studies of transporter expression.^196^ For example, EAAT1 mRNA was significantly reduced in the mediodorsal nucleus of the thalamus in an enriched population of astrocytes. In contrast, a significant increase in EAAT2 mRNA expression was found in a population of excitatory thalamic relay neurons.^195^ Differential dysregulation of glutamate transporter function in different cell types increases the complexity of the contribution of glutamate transporter dysfunction to the pathophysiology of disease.

Similar to findings in ALS, EAAT2b mRNA is increased in excitatory neurons in schizophrenia, including relay neurons in the mediodorsal thalamic nucleus and pyramidal neurons in the ACC.^195,197^ While the functional role of EAAT2b expression in neurons remains to be determined, it is tempting to predict that an increase in neuronal EAAT2b, rather than EAAT2a expression, increases localization of EAAT2b-containing isoforms of the EAAT2 protein complex near or at presynaptic or postsynaptic terminals. Immunostaining suggests that EAAT2 protein expression in neurons is increased in schizophrenia, although the contribution of EAAT2b to this finding is unclear. Since EAAT2b can transport glutamate at a similar rate to EAAT2 when expressed as a homotrimer,^46^ increased neuronal EAAT2b expression should not negatively impact glutamate transport and may act as compensation for reduced astroglial EAAT2 in disease.^198^ It is unclear if the unique PDZ-binding domain or specific trafficking mechanism of EAAT2b facilitates its increased neuronal expression in pathological conditions. Alternatively, increased neuronal EAAT2b could play a role in recycling glutamate directly from the synapse to compensate for deficiencies in the glutamate-glutamine transport cycle due to reduced astroglial EAAT2 expression in this illness.

### Other neuropsychiatric illnesses

There is evidence for reduced glutamate reuptake in Huntington’s disease in the striatum.^199^ EAAT2 mRNA was reduced in the neostriatum which correlated with disease severity.^200^ EAAT2 gene expression was also reduced in the ACC and DLPFC of depressed subjects.^201^ In a population of depressed subjects who committed suicide, astroglial transporters EAAT1 and EAAT2 were decreased in the DLPFC while neuronal transporters EAAT3 and EAAT4 were increased in ACC.^202^ EAAT2 levels were increased in the dentate nucleus of the cerebellum in essential tremor subjects but decreased in the cerebellar cortex compared to controls. Differential EAAT2 expression may be a mechanism to maintain excitatory/inhibitory balance in this disorder.^203^

Aberrant expression of EAAT2, particularly increased neuronal expression of EAAT2b, is a common feature of many neuropsychiatric disorders. The functional consequences are not fully understood and further work is required to derive the role of not only presynaptic expression of EAAT2, but the consequences of EAAT2b expression in disease.

## Conclusions

There is a solid and growing body of evidence directly implicating glutamate transporters in the modulation of excitatory transmission and the shaping of synaptic plasticity. Understanding of transporter function has expanded, from its original role as a sink for glutamate, to include a more fundamental role in shaping synaptic plasticity. Strong evidence supports the conclusion that spatial localization and temporal synchronization of EAAT2 activity with synapses facilitates the dynamic processes of excitatory transmission and neuroplasticity. It follows that perturbation of EAAT expression, localization, activity or regulation may have serious consequences for glutamate neurotransmission, leading to detrimental effects that may include non-physiological glutamate spillover, disturbing a finely-tuned synaptic modulatory system.

Interestingly, altered neuroplasticity is a core feature of schizophrenia and related disorders, resulting in gross pathological changes, circuitry deficits and cell level alterations in dendritic morphology. These changes are associated with cognitive dysfunction and other symptoms of these diseases. Does EAAT2 dysregulation play a central role in the disrupted neuronal excitability and plasticity seen in schizophrenia? Or are alterations in EAAT2 expression an epiphenomenon? Glutamate spillover, followed by activation of extrasynaptic glutamate receptors and diminished presynaptic activity offers a potential mechanism by which EAAT2 might contribute to the pathophysiology of disease states and in particular cognitive deficits found early in schizophrenia. However, to date, there is only robust evidence for glutamate transporter dysfunction by such a mechanism in animal models of addiction.

While animal models can provide mechanistic evidence, postmortem brain tissues are a useful translational substrate to test disease-specific changes in EAAT2 expression, localization, and activity. However, this substrate offers challenges for rigor and reproducibility, due to variability of subject demographics and tissue quality measures, as well as limited availability of material.^196^ Differences in tissue dissection protocols, postmortem intervals and prior medication exposure of subjects are potentially confounding factors that may contribute to observed variability in EAAT protein and mRNA expression data.^196^

Despite these limitations and with a few notable exceptions, reports of altered EAAT2 expression, particularly in regards to cell-specific and microdomain-specific alteration in splice variant expression, suggest significant dysregulation of the transporter expression in several neuropsychiatric illnesses. For example, altered EAAT2 expression in Alzheimer’s dementia is likely secondary to the gross structural changes in the brain, resulting from the massive loss of neurons associated with disease.^204^ In contrast, more subtle changes in EAAT2 expression are found in schizophrenia, which typically has a small reduction in astrocyte numbers with no change in the number of pyramidal neurons.^4^

In schizophrenia, the preponderance of genetic risk for this illness suggests “broken” synapses during development. We posit that changes in EAAT2 expression may represent an intermediate endophenotype, as the developing astroglial–neuron interface is dependent on bidirectional signals leading to the rich intertwined bioenergetic connections between these cells. We propose that cognitive decline seen early in schizophrenia might be a consequence of aberrant synaptic plasticity resulting from glutamate spillover due to alterations in EAAT2 expression and/or localization. However, additional experiments providing evidence for altered EAAT2 expression causing plasticity defects in animal models of schizophrenia (and other diseases) need to be performed, as the present data does not directly inform this hypothesis.

In summary, the glutamate transporters represent a critical biological substrate for excitatory neurotransmission. They provide a compelling target for understanding the pathophysiology of neuropsychiatric diseases, as well as the fundamental biology of synaptic plasticity. Accumulating evidence suggests that EAAT2 expression is altered in several diseases, and may have a direct role in the manifestation of clinical symptoms in substance use disorders. More work is needed, particularly with models of cognitive disorders, to develop these hypotheses, and to determine if there are substrates that may be exploited for the development of novel treatment strategies for these often devastating illnesses.
